# Scotch Physicians and Surgeons

**Published:** 1888-03-17

**Authors:** C. G. Furley


### Scotch Physicians and Surgeons.

By C. G. Furley.

(*Continued from p.* 381.)

### SIR ROBERT CHRISTISON.

About this time Professor Christison the elder—he of the Humanity class—died, leaving his family but slenderly provided for, as is the fate of professors of subjects whose practical bearing on success in life is not obvious. It seemed for a time as if this was destined to hinder his son Robert's progress; but, as with Simpson, there was a helpful elder brother—in this case a rising young lawyer—who put his means at the young doctor's disposal, and enabled him to complete his medical education by visiting the schools of London and Paris.

Richie Moniplies, it may be remembered, considered the Thames inferior to the Water of Leith. The unthinking will say that he lied when he magnified the river whose shallow waters run through his native town; the more charitable will perceive that he spoke the truth of things as he saw them; only, patriotism somewhat impaired the justness of his vision. Robert Christison suffered from a somewhat similar obliquity concerning Edinburgh University. It had its imperfections (when he was at home to see them); but it was vastly superior to any other school. Young Christison came to St. Bartholomew's, where, as he candidly says, he learned nothing; on the contrary, he found much to quarrel with. The students neglected medicine altogether, because it was a subject omitted from their examination; only three frequenting the medical wards to balance the hundreds who daily "walked" the surgical. The lecturers could teach nothing to Gregory's pupil, and the house-surgeons were less constant and conscientious in their attendance than those in Edinburgh. The curriculum is widened and the hospital management improved both at Bart's and at Edinburgh since 1820; but, nevertheless, it must appeal to the common-sense of the community that a man who means to practise both branches of the art of medicine should receive a diploma in each; and it is the nature of the average student to study most diligently the subject on which he must undergo the severest examination, even though his knowledge of it may be less called upon in after-life. A double qualification is practically essential to the general practitioner, and as the two branches of the profession belong to the same tree, will do no harm to the specialist as well.

From London Christison went to Paris, where he passed six months. Here, as in London, he got, he says, no lesson in the treatment of disease. Theories which could not be borne out by facts disputed the ground with old systems practised mechanically without any confirmation from fresh observations. The physicians seemed careless, the surgeons often stupid and always cruel. Among the latter there was one exception—Dupuytren, who of all "the faculty" in Paris had the most decided reputation for bearishness. His frankness to his patients was said to amount to brutality; but on the two occasions on which Christison had the opportunity of observing him, once going round the wards of a hospital, and once at the operating table, he was gentle and fatherly in manner. The young Scotchman was struck by this as much as by his surgical skill, and one cannot but admit the exceptional quality of a man who in pre-anæsthetic days could induce a child of seven to submit to the operation for stone without any other sign of the terrible pain than "a low moaning." Christison says it was remarkable to observe him going through his task with the speed that comes of assured skill, demonstrating to his crowd of students, and at the same time soothing and encouraging the little patient, who, when the operation was over, murmured a faint "Adieu, monsieur," to his benevolent torturer, to which the great surgeon replied gently, "Adieu, cher enfant." The bear who could achieve such a triumph as this must have had a very tender human heart behind his rough coat. Perhaps one of the things we have lost in gaining so many mechanical appliances for making life pass more easily is our knowledge of the resources and power of human sympathy and the human will. The best of us have begun to depend too much on outside aids; we have, whatever our creed, a too material habit of mind, and except in utmost extremity we make no use of soul power, that "nerve force" which even Herbert Spencer has to admit is yet uncatalogued by the most careful analysis.

One of the subjects of conversation in the intellectual circles of Paris in 1820 was the chemist Orfila, and those brilliant experiments with poisons which entitle him to be regarded as the founder of the science of Toxicology. Orfila did not lecture during the winter; so Christison did not hear him till April, 1821, only a few days before he left Paris. In view of this, it seems strange that none of his teachers should have had so great an effect on Christison's life-work as Orfila: he, too, will be longest remembered by his work in connexion with poisons. It may, however, have been to his advantage that he was not directly a pupil of the Frenchman; pupils are apt to be only echoes of their master's voice. He had, however, studied chemistry in Paris under Robiquet, a scientific chemist, as well as the owner of a "pharmacie," who taught him much in the way of care and accuracy in making investigations.

He would probably have spent more time in France in the prosecution of these chemical studies—for at this time his great desire was to go into the analysis of organic substances, a branch of study then prosecuted hardly at all in Britain—if he had not heard when there of the death of Gregory, which brought about a number of changes in Edinburgh University that finally left vacant the chair of Medical Jurisprudence. On hearing of his old teacher's decease Christison exclaimed regretfully, "He should have died later!" and went on with his work, thinking that he was too young for these changes to profit him. Medical Jurisprudence was a chair he would have liked to fill, but his youth and lack of experience seemed to make it unlikely that he would be chosen for it. The next news, however, was that his brother had put his name in the list of candidates for the post, and he came home to prosecute his canvass.

He had to do even more; he had to justify the existence of the chair. Medical jurisprudence was then taught nowhere in Britain except in Edinburgh, and being an optional subject and not very well taught, there was some question of giving it up. Christison had, in the first place, to demonstrate the necessity for retaining the chair, and then to secure it for himself. The struggle raged—there is no other word to describe the anxiety and rancour attending an Edinburgh professional election—for months, from May, 1821, to January, 1822; as usual, other considerations than the welfare of medical science affected the conflict, and it was by the aid of political influence that Christison gained the day.

It was at this time that there commenced his almost lifelong warfare with the Town Council of Edinburgh. The University of Edinburgh is no aristocratic foundation; it was known in its infancy as "Oor Toon's College"; it is altogether *bourgeois* in origin, and the municipality of the city had always had the right of appointing the larger number of its teachers. For some time during the last century the Lord Provost and magistrates had neglected to exercise their power, and then the medical school had increased greatly in power and usefulness. When, therefore, a new generation of town councillors desired to exercise their old jurisdiction, they found their undeniably lawful claim opposed by the University authorities. Endless quarrelling and hatred followed. The Council stood on their rights; the senators said harsh things about the incompetence of the average city magnate to choose the right man to fill a scientific post; and adult town and gown indulged in a more serious warfare than the street rows of southern university cities.

(*To be continued.*)

